# Construction of Prognostic Risk Model for Small Cell Lung Cancer Based on Immune-Related Genes

**DOI:** 10.1155/2022/7116080

**Published:** 2022-09-30

**Authors:** Feng Deng, Feng Tao, Zhili Xu, Jun Zhou, Xiaowei Gong, Ruhu Zhang

**Affiliations:** Thoracic Surgery Jiangyin Hospital Affiliated to Nanjing University of Chinese Medicine, Jiangsu, China

## Abstract

Small cell lung cancer (SCLC) is a highly invasive and fatal malignancy. Research at the present stage implied that the expression of immune-related genes is associated with the prognosis in SCLC. Accordingly, it is essential to explore effective immune-related molecular markers to judge prognosis and treat SCLC. Our research obtained SCLC dataset from Gene Expression Omnibus (GEO) and subjected mRNAs in it to differential expression analysis. Differentially expressed mRNAs (DEmRNAs) were intersected with immune-related genes to yield immune-related differentially expressed genes (DEGs). The functions of these DEGs were revealed by Gene Ontology (GO) and Kyoto Encyclopedia of Genes and Genomes (KEGG) enrichment analyses. Thereafter, we categorized 3 subtypes of immune-related DEGs via K-means clustering. Kaplan-Meier curves analyzed the effects of 3 subtypes on SCLC patients' survival. Single-sample gene set enrichment analysis (ssGSEA) and ESTIMATE validated that the activation of different immune gene subtypes differed significantly. Finally, an immune-related-7-gene assessment model was constructed by univariate-Lasso-multiple Cox regression analyses. Riskscores, Kaplan-Meier curves, receiver operating characteristic (ROC) curves, and independent prognostic analyses validated the prognostic value of the immune-related-7-gene assessment model. As suggested by GSEA, there was a prominent difference in cytokine-related pathways between high- and low-risk groups. As the analysis went further, we discovered a statistically significant difference in the expression of human leukocyte antigen (HLA) proteins and costimulatory molecules expressed on the surface of CD274, CD152, and T lymphocytes in different groups. In a word, we started with immune-related genes to construct the prognostic model for SCLC, which could effectively evaluate the clinical outcomes and offer guidance for the treatment and prognosis of SCLC patients.

## 1. Introduction

Small cell lung cancer (SCLC) represents 15% of all lung cancer (LC) cases. 60%-65% of SCLC patients had metastasis at diagnosis [1, 2]. SCLC grows fast with high invasiveness, which results in the poor prognosis of patients [3]. Presently, the combination of radiotherapy and chemotherapy is the standard to treat SCLC patients [3]. Nevertheless, over recent years, works have revealed that only some patients with limited-stage SCLC benefit from such a treatment pattern (41% and 16% for the 2- and 5-year survival, respectively), but this pattern does not work well for patients at extensive-stage (distant metastasis of cancer), with 6.4% of overall response rate after treatment [4]. The emergence of targeted therapy has improved the prognoses of lung cancer patients but has only limited effects on patients with SCLC. To take an example, the epidermal growth factor receptor (EGFR) inhibitor erlotinib is successful in treating non-small cell lung cancer (NSCLC) but is less effective in treating SCLC [5]. The reason is that EGFR is not an effective target due to the extremely low mutation frequency in SCLC. Imatinib, a KIT gene-targeted drug, can effectively improve the prognoses of NSCLC patients but is not effective on SCLC patients [6, 7]. Imatinib cannot be an effective drug for SCLC because KIT mutation rarely exists in SCLC [6, 7]. Accordingly, the results are less satisfactory in SCLC patients' prognoses by using present treatment methods. Now, it is essential to seek prognostic targets at the molecular level for clinicians to precisely assess the prognosis and guide the treatment of SCLC patients, thereby improving the poor prognoses of SCLC patients.

As reported, the malignancy of cancer is associated with the immune-infiltrating microenvironment apart from the regulation of tumor cells [8]. A report analyzed the connection between T-cells and clinical outcomes of tumor patients, revealing that the infiltration of lymph cells, mainly cytotoxicity T cells and memory T cells, are positively linked to favorable clinical outcomes in patients with malignancy [9]. At the same time, there are accumulating works proving that immune-related genes can either facilitate or suppress cancer by affecting the immune microenvironment in cancer. It is of great significance to find out immune-related markers at the molecular level. For instance, lncRNA KCNQ1OT1/CD155 axis facilitates antitumor immune response by regulating T-cell depletion status in colorectal cancer; and lncRNA KCNQ1OT1 level is an indicator of patients' prognoses by reflecting the immune status of tumor tissue [10]. Immune-related gene PDK1 is able to manipulate PD-L1 level in tumor tissue by mTOR signaling, and further affects the immune escape of tumor [11]. The tumor progression can effectively slow down by downregulating tumor immune escape via PDK1 [11]. Besides, traditional immune markers like PD-1/PD-L1/CTLA4 play a marked role in indicating cancer progression and drug guidance [12]. Given that, we posited that there was a connection between immune-related genes, immune infiltrating microenvironment in tumor tissue, and prognoses of patients with malignancies. And potential targets can be revealed for improving the clinical outcomes of these patients by investigating such a connection. To investigate, the immune-related regulation can effectively help with the development of clinical drugs and medication guidance.

We constructed a 7-gene model for assessment of the prognoses of SCLC patients based on immune-related genes in the GSE60052 dataset and ImmPort Shared Data (ImmPort). We divided samples into high- and low-risk groups based on their Riskscores. Differences in the expression of the immune-related proteins, human leukocyte antigen (HLA), and the immune checkpoint gene between SCLC high- and low-risk groups were revealed by Wilcox test to reveal the underlying function of the prognostic model in predicting the immune mode of samples. The biomarkers we herein revealed and the risk assessment model can be positive tools for SCLC patients' prognoses.

## 2. Materials and Methods

### 2.1. Data Resource

mRNA and clinical data from GSE60052 (platform: GPL11154) of SCLC were downloaded from the GEO database (Home-GEO-NCBI (http://nih.gov)), containing 7 healthy samples and 79 tumor samples. Gene data closely correlating cellular immune function were from the ImmPort.

### 2.2. Screening of Differentially Expressed Genes (DEGs) Related to Immune Regulatory Functions in Cells

The mRNA data of healthy and tumor samples in GSE60052 were differentially analyzed using the *R* package “limma” [13], and DEGs with |logFC| > 1.0 and FDR < 0.05 were retained. Immune-related DEGs were sieved by taking an intersection of DEGs and genes associated with immune regulation function in ImmPort. The Gene Ontology (GO) and Kyoto Encyclopedia of Genes and Genomes (KEGG) analyses were performed on immune-related DEGs using the R package “clusterProfiler” [14] (*p* value < 0.05).

### 2.3. Consensus Clustering and Subtype Assessment Based on Immune-Related DEGs

To identify the immune subtype of SCLC, K-means clustering algorithm [15] was performed on immune-related DEGs using the R package “ConsensusClusterPlus”. The overall level of immune infiltration in subtype groups was assessed using the R package “ESTIMATE” (https://bioinformatics.mdanderson.org/estimate/rpackage.html). The immune score, matrix score, ESTIMATE score, and tumor purity calculated were subject to Wilcox analysis to plot violin plots in different subtypes. The activation of immune-related gene sets in different immune subtypes was assessed using R package “GSVA” [16] based on single-sample gene set enrichment analysis (ssGSEA) method.

### 2.4. Construction and Validation of Prognostic Models Related to Immune Regulatory Functions in Cells

Using the R package “survival” (survival: Survival Analysis (http://r-project.org)), univariate Cox regression analysis was performed on immune-related DEGs and those with *p* value < 0.05 were selected as the candidate genes. To prevent model overfitting, Lasso regression analysis of candidate genes was performed using the R package “glmnet” [17], and cross-validation was used to choose penalty parameter *λ* to remove genes with strong correlation to reduce model complexity. R package “survival” (survival: Survival Analysis (http://r-project.org)) was utilized to construct a multivariate Cox regression model for candidate genes screened by Lasso regression analysis. Based on the following formula, the obtained characteristic genes and Riskscore were subject to cumulative weighting to generate the prognostic model:

(1) $$\begin{array}{c} \text{Riskscore}=\overset{n}{\underset{i=1}{\sum }}\text{e}\text{x}{\text{p}}_{i}*\beta_{i}, \end{array}$$

Where *n* is the number of genes related to immune characteristics and patient's prognosis, exp_*i*_ is the expression level of each gene, and *βi* is the risk coefficient calculated by multivariate Cox analysis.

The risk score of the patients was calculated according to the expression and risk coefficient of each gene in the prognostic model, and the samples were divided into high- and low-risk groups using the median Riskscore. By using R package “survival” (survival: Survival Analysis (http://r-project.org)), we plotted the survival curves of the high- and low-risk groups based on the Riskscore. R package “timeROC” plotted the ROC curves [18].

### 2.5. GSEA

KEGG pathway enrichment analysis was performed on high- and low-risk groups using GSEA software.

### 2.6. Analysis of Levels of Immune-Regulating Molecules in Tumor

Box plots were prepared by Wilcox analysis in each sample to count the expression of HLA proteins, immune checkpoints PD-L1, PD-1, CDLA4, and CD28.

### 2.7. Assessment of the Independence of the Prognostic Model

Univariate and multivariate regression analyses were performed on GSE60052 samples by combining patients' clinical information such as age, gender, stage, pathological stages (T and N), and prognostic Riskscores to draw the corresponding forest plot [19].

## 3. Results

### 3.1. Screening and Enrichment Analysis of Immune-Related DEGs

**Figure 1:**
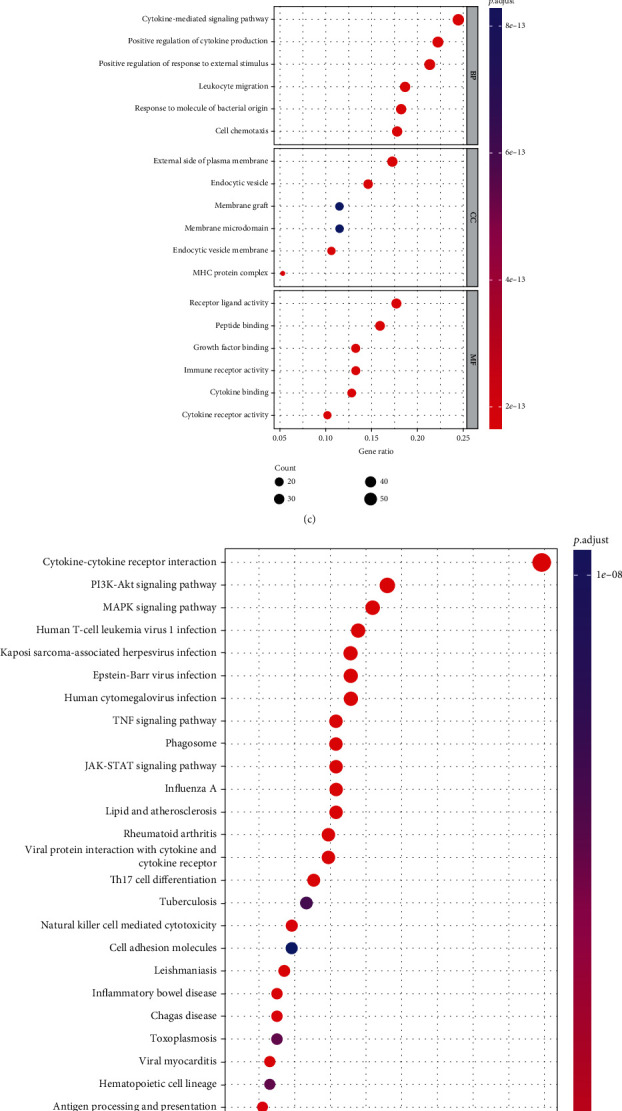
The screening and functional enrichment of immune-related DEGs. (a) Volcano plot of DEGs in SCLC samples and healthy samples in GEO dataset. Red represents significantly upregulated genes and green represents significantly downregulated genes. (b) Venn diagram of DEGs and immune-related genes. (c) GO and (d) KEGG analyses of immune-related DEGs.

Differentially expression analysis between normal and tumor groups in GEO data set yielded 3,022 DEGs in SCLC, including 1,301 highly expressed genes and 1,721 lowly expressed ones in tumor tissue (Figure 1(a)). Immune-related genes were searched from the ImmPort database. DEGs and immune-related genes were intersected to obtain 228 immune-related DEGs (Figure 1(b), Table S1). GO analysis suggested that these immune-related DEGs were mainly enriched in biological functions of positive regulation of cytokine production, positive regulation of response to external stimulus, and leukocyte migration (Figure 1(c)). KEGG analysis suggested that these DEGs were mainly enriched in PI3K-Akt signaling pathway, MAPK signaling pathway, and human T-cell leukemia virus 1 infection (Figure 1(d)).

### 3.2. Construction and Evaluation of SCLC Subtypes Based on Immune-Related DEGs

**Figure 2:**
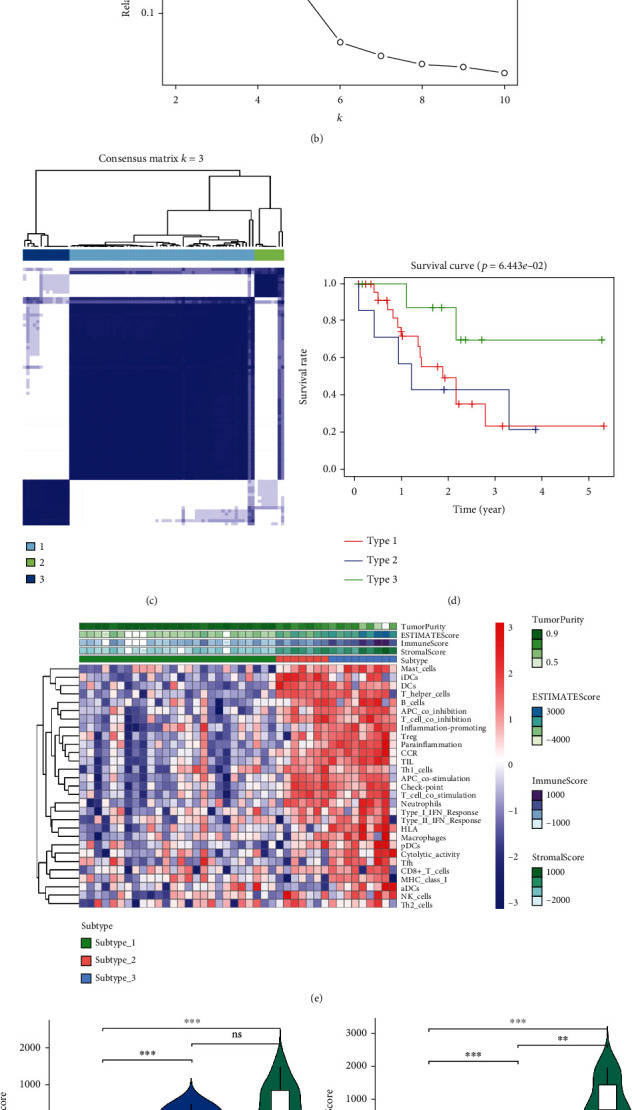
Construction and assessment of SCLC subtypes based on immune-related DEGs. (a) Diagram of consensus cumulative distribution function (CDF). (b) Plot of the relative area under the CDF curve. (c) K-means cluster analysis of SCLC samples. (d) Survival analysis of different immune subtypes. (e) Heatmap of tumor microenvironment in different subtypes. (f) Stromal score (red = subtype-1, blue = subtype-2, and green = subtype-3), (g) immune scores (red = subtype-1, blue = subtype-2, and green = subtype-3), (h) ESTIMATE scores (red = subtype-1, blue = subtype-2, and green = subtype-3), and (i) Tumor purity scores for different subtypes (red = subtype-1, blue = subtype-2, and green = subtype-3). ns: not significant; ^∗∗^*P* < 0.01; ^∗∗∗^*P* < 0.001.

Consensus clustering analysis was conducted on 228 immune-related DEG loci to identify molecular subsets of different immune-related DEGs. Clustering results were visualized using cumulative distribution function (CDF) plots and CDF incremental area plots, where *k* represents the number of groups (Figures 2(a) and 2(b)). The results of undifferentiated clustering analysis showed that the internal consistency of the clusters was high, and the clustering effect was best when *k* = 3 (Figure 2(c)), so the samples could be categorized into three subtypes. Survival curves were plotted by grouping the subtypes and combining clinical information for survival analysis, showing that subtype 3 had a higher survival rate than subtypes 2 and 1 (Figure 2(d)). Among them, the activation levels of immune function-related gene sets of subtype 2 and subtype 3 were higher than those of subtype 1 (Figure 2(e)). Further evaluation of the immune microenvironment of these three subtypes indicated that there were differences in the tumor microenvironment scores among the three subtypes. Of which, the subtype 3 with the best overall survival status had the highest stromal score, immune score, and ESTIMATE score and the lowest tumor purity, while the subtype 1 presented opposite results to that of subtype 3 (Figures 2(f)–2(i)). The above results indicated that the immune-related DEGs selected in this study can distinguish SCLC samples based on immune status and that there were marked differences in the prognosis among the three immune subtypes, indicating the feasibility of constructing prognostic model with these genes.

### 3.3. Prognostic Modeling and Validation of DEGs Associated with Immune Regulatory Functions in Tumor

**Figure 3:**
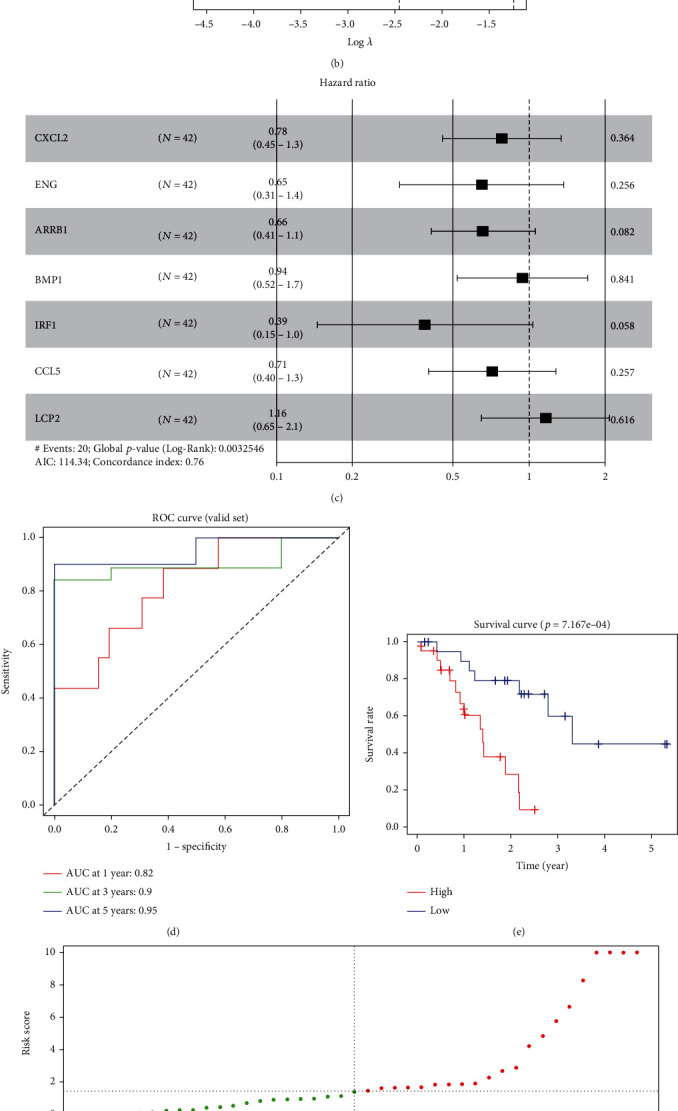
Construction of a prognostic risk assessment model for SCLC patient. (a) Trajectory plot of the gene coefficients with the increasing log (*λ*) value in the Lasso model. (b) The optimal log (*λ*) was selected according to the partial likelihood deviance in the Lasso model. (c) Forest plot of 7 genes obtained from multivariate Cox regression analysis. (d) ROC curves of the GSE60052 data sample risk score in both groups. (e) Survival analysis of GSE60052 dataset by the Riskscore model. (f) Distribution of scores, (g) survival status, and (h) the heatmap of 7 genes in both groups.

Univariate Cox regression analysis was performed on DEGs associated with immune function to obtain 20 candidate genes with prominent association with survival (Table S2). Lasso regression analysis of these 20 candidate genes filtered out high-fit immune function-related DEGs. As depicted in Figure 3(b), the smaller the partial-likelihood deviance value, the more stable the model was. When the log (*λ*) was 2.5, the model was relatively more stable, and thus, the log (*λ*) value was considered to be the optimal penalty coefficient. As plotted in Figure 3(a), the regression coefficients varied with log (*λ*). A multivariate regression model was established for these 7 signature genes associated with prognosis, and the final modeling was completed based on the risk score coefficient of each gene: Riskscore = −0.24923 × CXCL2 − 0.43238 × ENG − 0.41932 × ARRB1 − 0.06037 × BMP1 − 0.94496 × IRF1 − 0.33634 × CCL5 + 0.14949 × LCP2 (Figure 3(c)).

ROC curves for assessing the 1-, 3-, and 5-year overall survival of patients' prognosis were plotted based on the Riskscores obtained from this model, and their AUC values were 0.82, 0.9, and 0.95, respectively (Figure 3(d)). The survival curves were plotted by grouping the samples into high- and low-risk groups according to the median value of the Riskscore, and patients in the high-risk group were found to have lower survival (Figure 3(e)). Combined with Riskscore distribution and survival time, it was found that the increase of Riskscore was associated with the increased number of deaths and the decreased survival time of patients (Figures 3(f) and 3(g)). And there was a difference in the level of the 7 genes (Figure 3(h)). In sum, our work generated a 7-signature gene risk assessment model to predict the prognosis of SCLC patients, with good predictive ability.

### 3.4. KEGG Analysis of High- and Low-Risk Groups

**Figure 4:**
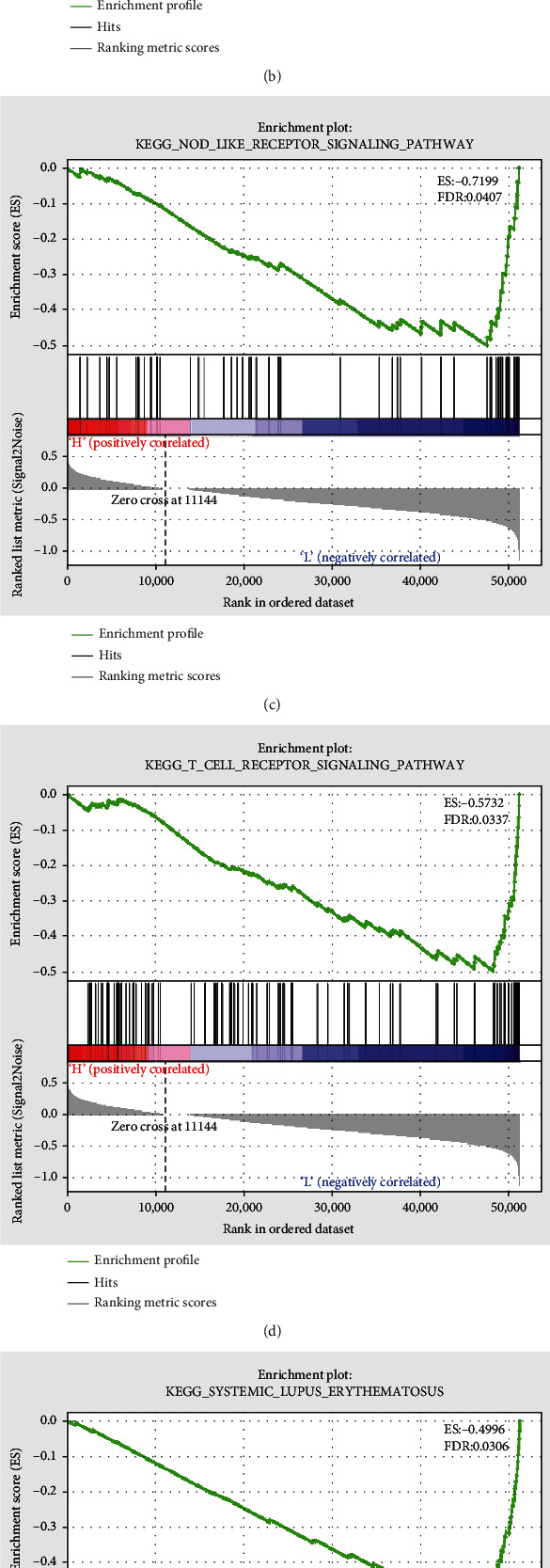
GSEA for high- and low-risk groups.

KEGG analysis of both groups using GSEA software revealed that the low-risk group was markedly enriched in immune-related pathways, including chemokine signaling pathway, FC gamma R mediated phagocytosis, nod-like receptor signaling pathway, T cell receptor signaling pathway, systemic lupus erythematosus, and B cell receptor signaling pathway (Figures 4(a)–4(f)). Most of the above pathways belong to cytokine-related pathways, which are closely related to tumor immunity and antitumor killing effect, supporting the difference in immune pattern between groups, and also implying that the difference in prognoses of patients in high- and low-risk groups in this study may be associated with these signaling pathways, but it still needs subsequent validation.

### 3.5. Analysis of Immune Characteristics in Tumor Based on the Risk Assessment Model

**Figure 5:**
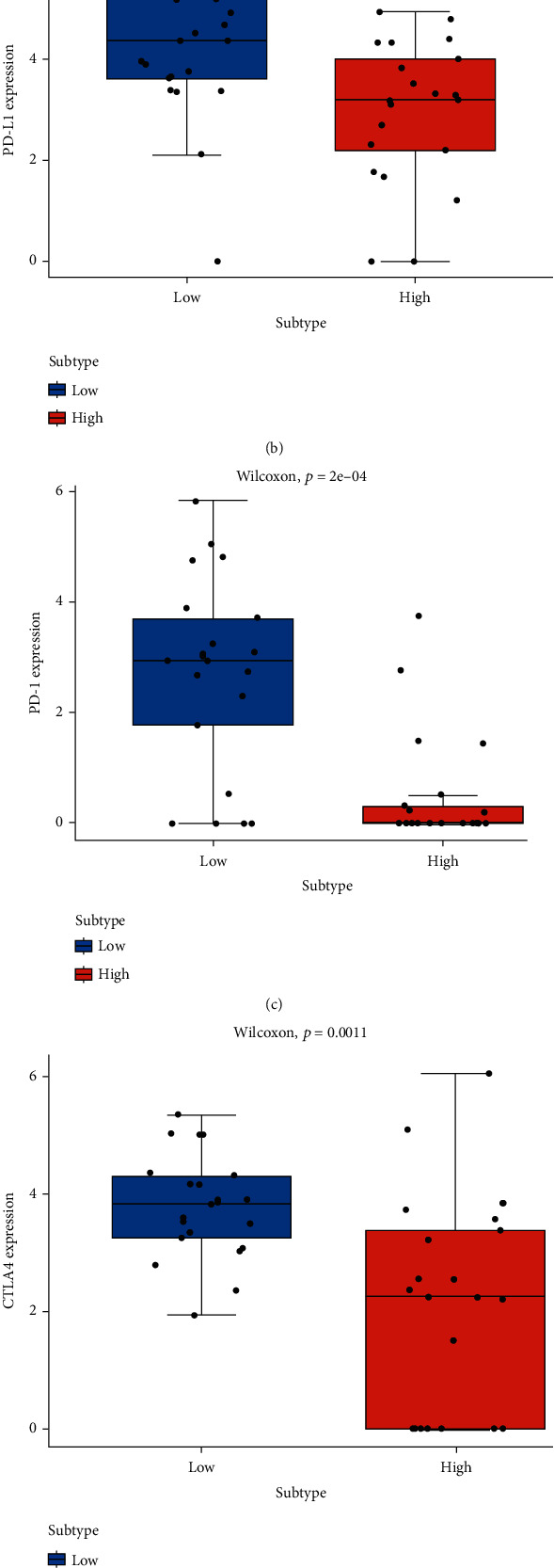
Analysis of tumor immune characteristics in high- and low-risk groups. Box plots of (a) HLA, (b) PD-L1, (c) PD-1, (d) CTLA4, and (e) CD28 in high- (red) and low-risk (blue) groups. ns: not significant; ^∗^*P* < 0.05; ^∗∗^*P* < 0.01; ^∗∗∗^*P* < 0.001.

Given that the GSEA elucidated the differences in immune signaling-related pathways between the high- and low-risk groups, we worked to further elucidate the differences in immune patterns between groups, HLA expression in the high- and low-risk groups. The results indicated significantly higher expression of HLA series antigen molecules in the low-risk group (Figure 5(a)). Immune-related checkpoint molecules or targeted inhibitory molecules of immunotherapy, PD-L1 (Figure 5(b)), CTLA4 (Figure 5(d)), and CD28 (Figure 5(e)) manifested statistically significant differences of high levels in the low-risk group. While the targeted inhibitory molecule PD-1 showed no significant difference in the low-risk group (Figure 5(c)). The above models validated the differences in immune patterns between high- and low-risk groups, and the differences in patient outcomes between both groups may be driven by differences in these immune proteins or checkpoints.

### 3.6. Evaluation of Clinical Value of the Prognostic Model

**Figure 6:**
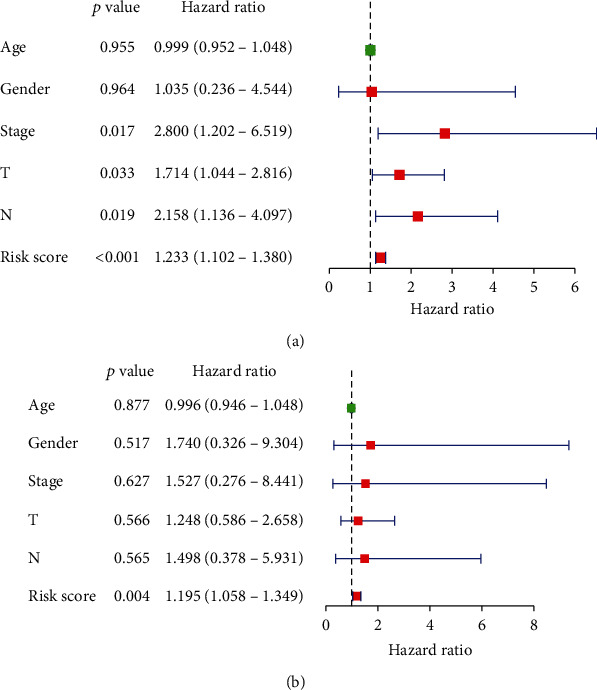
Correlation analysis between Riskscore and clinical factors in SCLC patients. (a) Forest plot of univariate and (b) multivariate Cox regression analyses based on clinical information, Riskscore, and overall survival.

Univariate regression analysis on Riskscore and clinical information revealed significance between tumor clinical stages, pathological stages (T and N) and Riskscore (*p* value < 0.05) (Figure 6(a)). Subsequent multivariate regression analysis revealed that significance only lay in Riskscore (Figure 6(b)). The prognostic score model constructed from these 7 signature genes could therefore be used as an independent prognostic factor.

## 4. Discussion

Tumor immune-related genes are important for revealing the prognosis of LC patients. CD133 is a key biomarker closely related to the prognosis of patients, and the use of this marker for tumor screening in SCLC can detect the presence of tumors earlier and reduce the risk of disease in patients [20]. At present, the use of bioinformatics approach to mine public databases and select effective therapeutic targets or biomarkers is currently the most advanced research method. As proposed by Wu et al. [21], a TME risk model constructed with the tumor immune infiltration-related genes SERPINE1, CX3CR1, CD200R1, GBP1, IRF1, STAP1, LOX, and OR7E47P based on public databases can be used to predict the prognostic survival of LC patients. Our study focused on exploring the correlation between SCLC immune-related genes and prognosis while distinguishing SCLC immune subtypes, revealing differences in immune-related molecular indicators between these subtypes, and validating the effectiveness of related models. Most importantly, this study constructed a risk assessment model based on immune-related DEGs that can be used to assess the prognoses of SCLC patients, which provided a basis for SCLC diagnosis and treatment.

In this study, we identified 7 genes that can be used to construct a prognostic model for SCLC patients via bioinformatics approach, of which CXCL2, ENG, ARRB1, BMP1, IRF1, and CCL5 were prognostic protective factors and LCP2 was the only prognostic risk factor. Among them, CXCL2 is a traditional inflammatory chemokine, which can mediate the recruitment of neutrophils to lung tissue [22]. This may provide a reference for CXCL2 as a prognostic risk factor in this paper; the recruitment of neutrophils by CXCL2 may facilitate antitumor immune responses. ENG (CD105) is a coreceptor of TGF-*β*, which is essential for angiogenesis/vascular development. The expression of this gene can impel angiogenesis in tumor tissue and further cause malignant progression of tumors, which has been always considered as an excellent therapeutic target [23]. There was a mice experiment indicating that anti-ENG monoclonal antibody in treatment can suppress tumor progression [24]. The possible effect of ARRB1 on tumor development is still controversial, and ARRB1 is associated with the prognosis of patients with tumors in a variety of cancers [25–27]. In our study, data models suggested that ARRB1 may improve the prognosis of patients with SCLC. BMP1 is considered to be a key factor in promoting tumor growth and metastasis of LC, and BMP1 facilitates NSCLC metastasis by inhibiting TGF-*β* activity in NSCLC [28]. Interestingly, it was found in the present study that BMP1 may improve the prognoses of SCLC patients, and the reasons for this need to be further elucidated. The expression of IRF1 in NSCLC tissue was generally lower than that in normal lung tissue, which is cancer-suppressive by regulating KPNA2 [29]. We found that IRF1 could be used as a prognostic protective factor in SCLC, consistent with the previous study. CCL5 acts as a chemokine ligand, which is supposed to propel the cytotoxicity of tissue-resident T and NK cells and strengthen antitumor immune responses [30]. LCP2, the only prognostic risk factor among the 7 genes of our prognostic model, is thought to be related to the infiltrating level of toxic lymphocytes and plays a regulatory role in antitumor immunity. Unfortunately, its role in SCLC remains unknown [31, 32]. In addition, in the study of LC, it has been exhibited that this gene is positively correlated with PD-L1 level in lung adenocarcinoma tissue, and we speculated that it may further affect the disease progression of SCLC by affecting the immune escape mechanism in cancer [33]. In short, the prognosis-related marker molecules associated with immune regulatory function obtained in this paper were generally involved in the regulation of tumor growth and immunity. These molecules can serve as biomarkers to predict the prognosis of patients and potential targets for SCLC treatment.

After establishing a prognostic model, we divided SCLC samples into high- and low-risk groups based on the median value of the risk score of the risk assessment model, and the results implied that the HLAs, especially HLA-DPA1, HLA-DPB1, HLA-DMB, and HLA-DOA, were remarkably upregulated in the low-risk group. HLA is the expression product of the major histocompatibility complex (MHC) in humans, and the HLA system is the most complex polymorphic system in the human body known so far. Given that this family is often dysregulated in the tissue of patients receiving immunotherapy, recent studies put their focus on the immune-regulatory effects of this family [34]. To take an example, HLA-DPA1and HLA-DPB1, dysregulated in our low-risk group, have been manifested to be associated with the maintenance of long-term immune efficacy after HBV vaccination [35]. In the field of cancer, HLA-DPA1 activates chemokines and toll-like receptor signaling pathways to regulate hepatocellular carcinoma progression [36]. HLA-DPB1 can be an antitumor factor to recruit NK cells, CD8^+^ T cells, and tumor-infiltrating lymphocytes such as Th1 and Tfh into breast cancer [37]. Both HLA-DPA1 and HLA-DPB1 play an antitumor role by activating immune cell infiltration, which well explained their downregulation in the high-risk group of this study. HLA-DMB is an essential component of MHC complex synthesis, and the expression of this gene is prominently positively correlated with the level of infiltration of tumor-infiltrating CD8^+^ T cells [38]. Similarly, HLA-DOA regulates the level of B cell infiltration in tissue and ensures the stable expression of MHC in cancer tissue, balancing their biological functions [39]. It can be seen that, similar to HLA-DPPA1 and HLA-DPB1, HLA-DMB, and HLA-DOA are also associated with the upregulation of tumor-infiltrating immune cells, consistent with the results predicted by our study. The immune-regulatory effect of HLA family validated the effectiveness of the construction based on the prognostic model of immune-related genes and also confirmed the rationality of this risk assessment model in predicting tumor immune patterns. In addition to leukocyte antigens, we observed notable differential expression of immune checkpoint genes PD-L1, CTLA4, and CD28 in high- and low-risk groups. Activated T cells often express PD-1 on their surface and can act as immune checkpoint receptors, while PD-L1 produced on the surface of many cancer cells acts as a PD-1 ligand and the combination of the two leads to tumor immunosuppression [40]. CTLA4 is an immune checkpoint protein expressed on activated T cells to downregulate the activation of T cells [41]. CD28 is a cell surface glycoprotein receptor expressed primarily on activated T cells and belongs to the immunoglobulin (Ig) superfamily [42]. This molecule has been manifested to negatively regulate T cell antitumor responses and is widely involved in tumor immune escape [42].

In summary, in this study, based on bioinformatics analysis, immune-related DEGs in the GSE60052 dataset were screened and classified into three subtypes that could represent different immune patterns to assess the rationality of immune-related genes. At the same time, the 7-gene prognostic model established by bioinformatics analysis based on immune-related DEGs could evaluate the prognoses of SCLC patients more accurately. The GSE60052 dataset was divided into high- and low-risk groups according to the median Riskscore, revealing the differences in the expression of immune checkpoint genes and antigen molecules, and confirming the rationality of the model in predicting tumor immune patterns. This study is conducive to deepening the understanding of immune-related genes in SCLC while providing a powerful tool for prognostic evaluation and immunotherapy of SCLC patients. Of course, this study has some limitations; the data of this study were derived from open databases, there were certain systematic errors, and the accuracy of model prediction needs to be subsequently verified in more clinical samples. Additionally, wet experiments were not conducted to validate the constructed model. Relevant cellular experiments and molecular experiments are therefore warranted to verify the model.

## Data Availability

All data generated or analyzed during this study are included in this article. Further enquiries can be directed to the corresponding author.

## Supplementary Materials

Supplementary 1 Table S1 Screening of 228 immune-related differential genes.

Supplementary 2 Table S2 Univariate Cox regression analysis of immune-related DEGs.
