# Supplementary material for: Construction of Prognostic Risk Model for Small Cell Lung Cancer Based on Immune-Related Genes
Source: Comput Math Methods Med. 2022 Sep 30;2022:7116080. doi: 10.1155/2022/7116080 (PMC9554662; doi:10.1155/2022/7116080)
Supplement: Supplementary 1 — Table S1 Screening of 228 immune-related differential genes. [file 7116080.f1.pdf]

Common elements in DE_gene immune_gene :
AGER
NFKBIA
PGC
ADRB2
DUOX1
CXCL2
ICAM1
A2M
TGFBR2
AHNAK
EDN1
ACVRL1
SFTPD
IL4R
SFTPA2
CSF3
SFTPA1
ADRB1
MARCO
TNFAIP3
S1PR1
CAT
ENG
SOCS3
FOS
AREG
HLA-E
SDC4
IL1RL1
FPR1
VIPR1
CSF3R
FGFR4
LRP1
ELN
CX3CL1
EDNRB
EGFR
STAT3
PTGDS
FABP4
CD74
SLC11A1
HLA-DPA1
FLT4
CD81
ARRB1
ZYX
MAP3K8
CRIM1
GRN
HLA-DRA
LTBP2
CSF1
ELAVL1
DMBT1
PDGFRB

PRF1
NFAT5
IL18R1
IL7R
VIM
BMP1
PTGS2
TIE1
NAMPT
C3
IL16
TEK
HLA-DMA
GPR17
NFKBIZ
BIRC5
IL6R
CSRP1
IL32
PDGFRA
CCL4
AQP9
NR4A1
CCL2
OLR1
TNFRSF10B
PDGFB
THBS1
RARA
ILK
CXCL3
OSMR
TLR2
IL6
FGR
NPR1
S100A10
SEMA3G
TNFRSF10D
SLPI
KDR
TFR2
SEMA5A
CXCL16
SEMA3B
NR3C2
CALCRL
CRLF3
BMP2
PML
TNFRSF1A
PTH1R
HDAC1
VGF
VEGFC
PPARG
HLA-DPB1
LCN12

RAC3
LTBP4
TRIM27
HBEGF
GDF10
KL
PTGER4
TGFB1
HLA-DRB1
TGFBR3
IL33
IRF1
COLEC12
MSR1
S100A11
IL1R1
CHP1
ICAM2
CCL3
CCL18
CD14
TINAGL1
LYN
CHGB
TXLNA
ITGAL
FURIN
TNFSF13
C5AR1
JUND
CIITA
CCR1
CTSE
TMSB15B
HLA-DOA
CXCR5
INPP5D
MICA
PIK3R1
NPR3
FCER1G
PDGFA
SEMA4A
BCL3
IL18RAP
CRHR2
SLC40A1
CD40
TRAJ37
PLXNA2
IL27RA
FLT1
TLR3
DES
TNFRSF1B
EDNRA
SDC1
NR3C1

CCL5
GNLY
ESRRG
SCGB3A1
IL17RB
TNFRSF25
ADIPOR2
FAS
HLA-B
SHC1
TNFSF12
B2M
LCP2
GNRH2
S100A16
RBP1
PTPRC
IL20RA
OPRD1
GHR
NRP1
ITGB2
LEPR
NFKB1
SCTR
MDK
S100A9
TLR4
TCF7L2
ABCC4
JAK1
SLIT2
DLL4
HLA-A
HLA-C
JUN
IL15RA
ISG20
FGF9
IFNGR1
OPRK1
ITGAV
SERPINA3
CTSB
CSF1R
ANXA6
CTSS
PTAFR
TNFRSF14
IL6ST
FGFR2
TNFRSF10A
IL13RA1
PAK6
CSF2RB
