# Supplementary material for: Construction of Prognostic Risk Model for Small Cell Lung Cancer Based on Immune-Related Genes
Source: Comput Math Methods Med. 2022 Sep 30;2022:7116080. doi: 10.1155/2022/7116080 (PMC9554662; doi:10.1155/2022/7116080)
Supplement: Supplementary 2 — Table S2 Univariate Cox regression analysis of immune-related DEGs. [file 7116080.f2.pdf]

gene	HR	z	pvalue
CXCL2	0.688753127	-2.557669271	0.010537626
TNFAIP3	0.664145138	-1.963245329	0.049617675
ENG	0.497450386	-2.630573986	0.008524081
LRP1	0.40430565	-2.349639495	0.018791601
CD74	0.685082383	-2.133534353	0.032880917
ARRB1	0.622874482	-2.579373798	0.009897962
CRIM1	0.666939767	-2.188693939	0.028619093
PRF1	0.748648651	-2.206341215	0.027360118
BMP1	0.56877346	-2.803136433	0.005060826
C3	0.697572447	-2.439468465	0.014708886
IL32	0.729442095	-2.208474484	0.027211215
IRF1	0.304587129	-2.680805806	0.007344512
CCL3	0.757852291	-2.125812168	0.033518902
ITGAL	0.776090016	-2.198468435	0.027915742
C5AR1	0.782843878	-2.039628813	0.04138731
CIITA	0.496688274	-2.494102436	0.01262761
TNFRSF1B	0.737601115	-2.661301599	0.00778392
CCL5	0.494157317	-3.049984897	0.002288529
LCP2	0.722795893	-2.728565262	0.00636105
CSF2RB	0.787575846	-2.243717988	0.024850552
